# Microbial- and thiosulfate-mediated dissolution of mercury sulfide minerals and transformation to gaseous mercury

**DOI:** 10.3389/fmicb.2015.00596

**Published:** 2015-06-23

**Authors:** Adiari I. Vázquez-Rodríguez, Colleen M. Hansel, Tong Zhang, Carl H. Lamborg, Cara M. Santelli, Samuel M. Webb, Scott C. Brooks

**Affiliations:** ^1^School of Engineering and Applied Sciences, Harvard UniversityCambridge, MA, USA; ^2^Department of Marine Chemistry and Geochemistry, Woods Hole Oceanographic InstitutionWoods Hole, MA, USA; ^3^Department of Mineral Sciences, Smithsonian Institution, National Museum of Natural HistoryWashington, DC, USA; ^4^Stanford Synchrotron Radiation LightsourceMenlo Park, CA, USA; ^5^Environmental Sciences Division, Oak Ridge National LaboratoryOak Ridge, TN, USA

**Keywords:** mercury, metacinnabar, sulfur chemosynthesis, *Thiobacillus*, thiosulfate, mercury sulfide dissolution, sulfur metabolism, sulfur oxidation

## Abstract

Mercury (Hg) is a toxic heavy metal that poses significant environmental and human health risks. Soils and sediments, where Hg can exist as the Hg sulfide mineral metacinnabar (β-HgS), represent major Hg reservoirs in aquatic environments. Metacinnabar has historically been considered a sink for Hg in all but severely acidic environments, and thus disregarded as a potential source of Hg back to aqueous or gaseous pools. Here, we conducted a combination of field and laboratory incubations to identify the potential for metacinnabar as a source of dissolved Hg within near neutral pH environments and the underpinning (a)biotic mechanisms at play. We show that the abundant and widespread sulfur-oxidizing bacteria of the genus *Thiobacillus* extensively colonized metacinnabar chips incubated within aerobic, near neutral pH creek sediments. Laboratory incubations of axenic *Thiobacillus thioparus* cultures led to the release of metacinnabar-hosted Hg(II) and subsequent volatilization to Hg(0). This dissolution and volatilization was greatly enhanced in the presence of thiosulfate, which served a dual role by enhancing HgS dissolution through Hg complexation and providing an additional metabolic substrate for *Thiobacillus*. These findings reveal a new coupled abiotic-biotic pathway for the transformation of metacinnabar-bound Hg(II) to Hg(0), while expanding the sulfide substrates available for neutrophilic chemosynthetic bacteria to Hg-laden sulfides. They also point to mineral-hosted Hg as an underappreciated source of gaseous elemental Hg to the environment.

## Introduction

Mercury (Hg) is a toxic heavy metal that poses significant environmental and human health risks (Wiener et al., 2002; Clarkson and Magos, 2006). Because gaseous elemental Hg travels over hemispheric scales, it is now an internationally recognized priority pollutant in need of global regulation (McNutt, 2013). Mineral associated Hg is the largest Hg reservoir in the environment where it can account for nearly 60% of the total Hg mass inventory (Mason et al., 2012). A large fraction of this pool is comprised of mercury sulfide minerals (HgS) (Smith-Downey et al., 2010).

The dominant Hg minerals are the two HgS polymorphs cinnabar (α-HgS) and metacinnabar (β-HgS). Metacinnabar is the dominant authigenic phase, where it forms under sulfidic conditions within soils and sediments (Barnett et al., 1997). Under acidic pH conditions (pH~3), HgS dissolution can be enhanced in the presence of mine tailing-derived microbial cultures (Jew et al., 2014). Under these acidic conditions, bacteria of the genus *Acidithiobacillus* have been implicated in inducing cinnabar dissolution and volatilization of released Hg (Baldi and Olson, 1987). Yet under non-acidic conditions (pH > 4), both HgS phases are considered to be poorly reactive and stable due to their low solubility (e.g., logK*_so_* ~ -39) (Dyrssen, 1989) and very slow abiotic dissolution kinetics (Barnett et al., 2001; Holley et al., 2007). There is a growing appreciation, however, for the role of dissolved organic matter in increasing HgS dissolution and inhibiting HgS precipitation and aggregation in the absence and presence of bacterial activity (Ravichandran, 2004; Graham et al., 2012). Nevertheless, HgS minerals are still largely considered as stable Hg phases and microbial-assisted dissolution of HgS at near neutral pH conditions is assumed to be negligible.

Observations of substantial Hg fluxes from unknown sources within terrestrial and marine sulfidic sediments, however, have called into question the validity of this assumption. For instance, HgS minerals have recently been implicated as a source of high dissolved Hg concentrations originating from sulfide-rich deep-sea vent sediments, raising the possibility that chemosynthetic microorganisms mobilize Hg from these low solubility phases (Crespo-Medina et al., 2009). Despite the widespread presence of HgS minerals in both terrestrial and marine sediments, a clear understanding of the extent and mechanisms of HgS dissolution in these systems is lacking. Here we address this knowledge gap by conducting a combination of field and laboratory incubations to identify the potential for metacinnabar as a source of dissolved Hg within near neutral pH environments and the underpinning mechanisms at play.

## Materials and methods

### Field incubations

Metacinnabar and pyrite specimens were obtained from the Harvard Museum of Natural History (HMNH) Mineralogical Collection (specimen #122749). For *in-situ* creek incubations, minerals were cut into sections approximately 2 cm by 2 cm by 1 mm and polished. Mineral sections were mounted on glass slides (2 cm by 2.5 cm), which were then secured into precut holes on a polycarbonate sampler, and covered in mesh to prevent large organisms from interfering with the slabs. This sampler was inserted directly into the East Fork Poplar Creek (EFPC) sediments at a depth of 2.5–5 cm in Oak Ridge, Tennessee, USA at N 36° 00.101', W 084° 15.011', ±18 ft. Minerals were incubated for 6 weeks between September and October 2010 within the aerobic sediments of the EFPC creek channel. The geochemistry of the EFPC porewater near sampler deployment location (EFK 22) has been extensively monitored, and Supplementary Table S1 includes geochemical parameters collected on the day of sampler retrieval.

Once retrieved from the creek, mineral samplers were transported on ice to our laboratory where they were preserved within 24 h. Mineral sections used for DNA extraction and sequencing were stored aseptically at −80°C. Minerals for thin sections were fixed using 4% paraformaldehyde, rinsed twice in PBS solution, and stored at −20°C in a 1:1 (v/v) mixture of PBS and 96% ethanol until further processing.

### Molecular methods

Field-incubated minerals were aseptically crushed and DNA was extracted using the Ultraclean soil DNA kit (Mo Bio Laboratories) using the maximum yield protocol with the following modifications. After minerals were added to the bead solution tubes, tubes were sonicated for 5 min. Following addition of IRS solution, tubes were incubated at 70°C for 10 min, and 200 μg of polyadenylic acid were added (Webster et al., 2003; Santelli et al., 2008), followed by vortexing at maximum speed for 15 min. The 16S rRNA region of environmental DNA was amplified using the 8F and 1492R primer set and conditions used previously (Lane, 1991; Turner et al., 1999; Hansel et al., 2008) in triplicate or quadruplicate to yield sufficient DNA for pyrosequencing. Amplification products were purified using the QIAquick nucleotide removal kit (Qiagen). Bacterial tag-encoded pyrosequencing (bTEFAP) was conducted by the Research and Testing Laboratory, Lubbock, TX using the GS FLX Titanium sequencing platform (Roche Applied Science). Primers 28F (5′GAGTTTGATCNTGGCTCAG) and 519r (5′GTNTTACNGCGGCKGCTG) were used to sequence variable regions V1-V3 of the 16S rRNA gene.

### Sequence processing

Pyrosequencing reads were denoised using AmpliconNoise, and chimeras were removed using Perseus (Quince et al., 2011). Following denoising, the metacinnabar and pyrite sequence library contained 10 846 and 5 177 sequence reads respectively with an average read length of 342 bp. Resulting sample reads were all longer than 150 bp, had a quality score greater than 20, and no ambiguous reads, therefore no further quality filtering was needed. Denoised, single reads were aligned and clustered using the Ribosomal Database Project (RDP) Pyrosequencing Pipeline (Nawrocki et al., 2009) and classified using a naïve Bayesian rRNA classifier, version 2.0, with a bootstrap cutoff of 80% (Wang et al., 2007). Eighty-two percent of denoised metacinnabar reads and 35% denoised pyrite reads were classifiable down to the genus level. Genus level abundances are presented as percent abundance of sequences falling within that genus relative to the total number of sequences classifiable at the genus level.

### *merA* gene amplification and sequencing

Genomic DNA from *T. thioparus* pure cultures was extracted using the Ultraclean soil DNA kit (Mo Bio Laboratories). A 285 bp fragment at the 3′ end of *merA* was amplified using PCR primers A1s-n.F and A5-n.R following previously reported amplification conditions (Chadhain et al., 2006). Gel electrophoresis was used for size separation of the PCR products, and the expected 285 bp *merA* amplification product was confirmed. The gel purified (QIAquick gel extraction kit, Qiagen) 285 bp amplification product was concentrated (DNA Clean and Concentrator-5, Zymo Research), cloned (StrataClone PCR Cloning Kits, Agilent Technologies) and sequenced. The nucleotide sequence of the *T. thioparus* partial *merA* sequence was analyzed by BLASTN 2.2.31 (Altschul et al., 1997) using the NCBI Genomic Reference Sequences (refseq_genomic) database.

### X-ray absorption spectroscopy

Fixed field-incubated metacinnabar slabs were air dried and embedded in EpoHeat Epoxy (Buehler). Cross sections (~500 μm) of the embedded minerals were obtained using a diamond saw, subsequently attached to a high-purity fused quartz slide using Hillquist Thin Section Epoxy A-B (Hillquist), and filed and polished down to 50–100 μm thickness using a microtome and 1000 grit silicon carbide paper (Buehler).

Spatially-resolved (μ-scale) X-ray fluorescence (XRF) and X-ray absorption spectroscopy were conducted at the Stanford Synchrotron Radiation Laboratory (SSRL) by collecting spectra at select points of interest or defining and rastering a defined region. Synchrotron-based μ-XRF on beamline 14-3 at SSRL was used to map the Hg (M-edge) and S (K-edge) spatial distribution within the mineral matrix and oxidation rind. Similarly, beamline 2–3 was used to map the distribution of Fe (K-edge) in relation to Hg. The beam size on the sample at both beamlines was approximately 2 × 2 μm. Total Hg and S distributions were collected at 2495 eV (BL14-3) and 13200 eV (BL2-3). Maps were also collected at several discrete incident energies in continuous raster scanning mode in order to collect the fluorescence at several distinguishing points within the S (2470.7, 2473, 2473.7, 2478.5, 2481.3, and 2483 eV) and Fe absorption edge (7123, 7126, 7128, 7130, 7133 eV) at beamlines 14-3 and 2-3, respectively. X-ray absorption near edge structure (XANES) spectra were collected at spots of interest to confirm the oxidation state at discrete locations and compared to standard spectra. Fluorescence maps and XANES spectra were analyzed using the MicroAnalysis Toolkit (Webb, 2006) and SIXPACK (Webb, 2005), respectively.

For bulk S solid-phase speciation, dried samples were mounted onto S-free Lexan. S XANES spectra were collected within a He-purged sample chamber under a continuous He flow with a Lytle detector at SSRL beamline 4-3. The spectra were calibrated with a thiosulfate standard. Samples were run in a He-purged anaerobic bag surrounding the sample holder chamber within the beamline hutch. XAS scans were averaged, background-subtracted, normalized, and deglitched if necessary using SIXPACK (Webb, 2005). The lineshapes (peak position and peak shape) of the XANES spectra were used to compare the relative proportions of different sulfur species within the sample.

### Pure culture incubations

Glass serum vials were soaked in 10% Instra-Analyzed HCl (J.T. Baker), and rinsed 4× in nanopure water. Glass vials were not reused following contact with Hg. Vials were loosely capped using similarly acid washed polypropylene caps which allowed air penetration while maintaining a sterile environment. Following contact with Hg caps were cleaned using a previously established protocol (Hammerschmidt et al., 2011). In order to close the system for volatile Hg capture, glass serum vials were capped with rubber stoppers and stainless steel needles were used to direct incoming and outgoing air. All incubation materials were autoclaved prior to use.

*Thiobacillus thioparus* (ATCC 8158) was incubated aerobically at room temperature in 100 mL glass serum vials in the dark for all experiments in the presence and absence of ground natural metacinnabar. Metacinnabar obtained from the HMNH Mineralogical Collection was sieved (<250 μm) prior to use in incubations, and surface area of processed minerals was determined using the BET Kr adsorption technique (0.1321 ± 0.0007 m^2^ g^−1^).

*T. thioparus* cultures were pre-grown in a pH 7 buffered basal freshwater medium containing 20 mM Na_2_S_2_O_3_, 29 mM KH_2_PO_4_, 23 mM K_2_HPO_4_, 3.8 mM Na_2_CO_3_, 7.5 mM NH_4_Cl, 1.0 mM MgCl_2_·6H_2_O, 0.3% vitamin solution (Widdel and Bak, 1992), and 0.1% Trace Element Solution (Widdel and Bak, 1992) at 30°C for 5 days. These cells were then centrifuged (5000× g for 10 min at 4°C) and concentrated approximately 30× by re-suspending in spent medium. A 1:100 inoculation of these cells was used for 40 μM, 60 μM, 100 μM and 20 mM initial thiosulfate experiments. For 20 mM initial thiosulfate experiments, a stock solution of sterile Na_2_S_2_O was prepared and added into incubations. For 40–100 μM initial thiosulfate experiments, no additional thiosulfate was added and the thiosulfate present was carryover from the inoculum, which served as a low level thiosulfate addition in the experiment. For zero thiosulfate experiments, centrifuged cells were re-suspended in thiosulfate-free medium, centrifuged again and then concentrated approximately 30× in the thiosulfate-free medium. A 1:100 or 1:50 inoculation of these cell suspensions was then performed.

Basal freshwater medium containing EDTA was used only for one sample series of aqueous Hg measurements as a function of thiosulfate concentration within incubations containing killed cells and metacinnabar. All other experiments were conducted in the absence of EDTA. Basal freshwater medium containing EDTA was identical to the basal freshwater medium described with the exception that Trace Element Solution was prepared following the T2 Medium for *Thiobacillus* Trace Metals solution (Atlas, 2005) using Na_2_MoO_4_ instead of (NH_4_)_2_MoO_4_. This Trace Element Solution contained 5% w/w EDTA (170 mM) resulting in 0.005% or 170 μM EDTA in the final medium.

Killed controls consisted of autoclaved (121°C, 30 min) concentrated cells or 6-day old pre-grown cultures that were killed with 4% formaldehyde, frozen overnight, thawed, centrifuged down, and re-suspended in thiosulfate-free medium. Similar to the processing for live cells, for killed cell preparations, the washing step was performed once for 40 μM and 20 mM initial thiosulfate experiments, and twice for zero thiosulfate experiments. Experimental inoculum contained elemental sulfur resulting from pre-growth in thiosulfate medium, and was homogenized to minimize differences in this carryover in the experiments. HgS used in experiments was sterilized by autoclaving under an oxygen-free N_2_ atmosphere to prevent mineral oxidation. Mineral loading used in all incubations with metacinnabar was 2 g L^−1^. Cultures were grown without shaking although vials were swirled prior to aseptically drawing samples for aqueous analyses.

Hg volatilization rates were determined by pumping air into *T. thioparus* incubations at a rate of 1.60 ± 0.2 mL min^−1^, and then capturing volatile Hg in outflow using gold-coated quartz sand traps. Incoming air was Hg-stripped using an upstream gold-coated sand trap and filtered through a 0.22 μm cellulose filter. Similarly, outflow was filtered upstream of the gold-coated quartz trap. The mass of Hg on traps was subsequently quantified by dual-stage gold amalgamation and the Tekran-2600 cold vapor atomic fluorescence spectrophotometer following previously established modifications (Lamborg et al., 2012). Hg emissions from incubations were sampled in intervals of minutes to hours to collect a Hg mass within the instrument detection range. Total Hg emissions from incubations were then estimated based upon volatilization rates obtained at time intervals throughout the experiment assuming a constant rate of change in volatilization rates. Hg emissions from incubations were sampled more frequently within the first few days of the experiment, with sampling frequency decreasing as the experiment progressed. Hg emissions from incubations started with no exogenous thiosulfate and 40 μM thiosulfate, were sampled daily for 2 and 7 days following inoculation. Sampling frequency was gradually decreased, with weekly sampling by day 19, and every 2 weeks by day 45. Incubations started with 20 mM thiosulfate were sampled daily for 4 and 9 days following inoculations with sampling frequency thereafter reduced to every 10 days.

For comparisons of Hg volatilization rates between natural and synthetic metacinnabar, a commercial synthetic metacinnabar (black HgS, Alfa Aesar) was used. The surface area of this synthetic metacinnabar was 1.2804 ± 0.0045 m^2^ g^−1^ as determined by BET Kr adsorption. The same natural metacinnabar used for other incubations was used for comparison. Live cells and incubations were prepared identically to the 40 μM thiosulfate incubations. Killed controls were autoclave killed cells. Incubations were spiked with a 25 mM thiosulfate stock solution every 24 h resulting in a daily 125–132 μM thiosulfate spike, with the range in concentration resulting from the volume change throughout the incubation (this is henceforth referred to as the 100 μM thiosulfate spike). Hg emission rates were quantified 0–16 h following the thiosulfate spike, and are presented as ng Hg per liter of culture volume per day.

Samples to be used for sulfate and thiosulfate quantification were filtered through a 0.22 μm cellulose filter and stored at −20°C until analysis. Sulfate and thiosulfate concentrations were quantified via suppressed anion chromatography with conductivity detection using a Dionex ICS-2000 (AS11 Column) with a KOH eluent generator. An eluent gradient method was employed (flow rate 1.5 mL min^−1^): beginning for 8 min at 1 mM, followed by a linear ramp to 15 mM over 4 min, another linear ramp to 60 mM over 8 min, followed by a sustained 60 mM for 2 min, and 1 mM for 13 min. A blank was run between all samples and standards to prevent carryover between samples. Samples for total dissolved Hg analysis were oxidized with 1–2% (v/v) bromine monochloride (BrCl) per EPA Method 1631. High thiosulfate samples had greater reducing capacity and were oxidized with either 5 or 10% (v/v) BrCl. Samples for total dissolved Hg analysis were refrigerated between collection and analysis except during the BrCl oxidation step. Calibration of the DMA-80 direct Hg analyzer was performed with a series of dissolved Hg(II) standards and the calibration was regularly verified using reference material from the Quebec National Institute for Public Health (INSPQ) Interlaboratory Comparison Program for Metals in Biological Matrices.

For the determination of total cell abundance, cultures were fixed with a final concentration of 4% formaldehyde and frozen. Prior to enumeration using epifluorescence microscopy, cells were stained using SYBR Green I Nucleic Acid Stain. A 0.1% p-phenylenediamine mounting solution was used to prevent photo bleaching (Noble and Fuhrman, 1998). Cell counts were performed for 20 random fields of view. Viable cell enumeration was conducted by making serial dilutions of the culture in PBS, and then plating onto agar dishes with the same culture medium.

## Results and discussion

### Colonization and oxidation of metacinnabar in creek sediments

**Figure 1:**
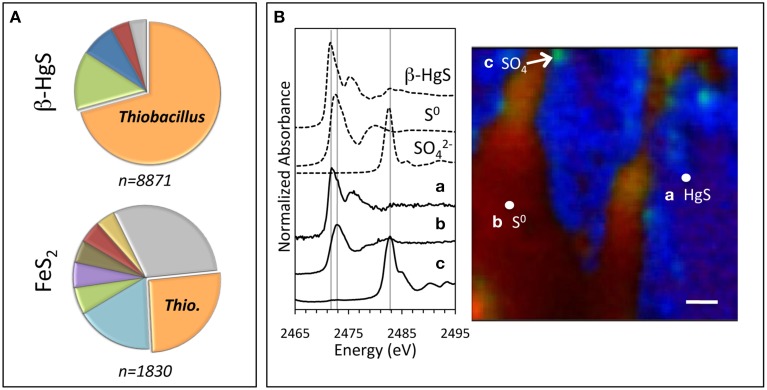
**Microbial and mineral composition of incubated mineral sections**. Sections were incubated within aerobic hyporheic sediments of the East Fork Poplar Creek for 6 weeks 2.5–5 cm below the sediment-water interface. **(A)** Genus-level classification of 16S rRNA sequences obtained from bacteria colonizing the metacinnabar (top) and pyrite (bottom) mineral sections. Pie chart percentages represent the number of sequences assigned to each genus divided by the total number of classified sequences, which include the known sulfur-oxidizing organisms *Thiobacillus* (orange, β-HgS = 71%, FeS_2_ = 26%), *Sulfuricurvum* (green, β-HgS = 13%, FeS_2_ = 6%), and *Sulfuricella* (dark blue, β-HgS = 8%), and also the bacterial genera *Methyloversatilis* (red, β-HgS = 4%, FeS_2_ = 5%), *Hyphomicrobium* (light blue, FeS_2_ = 17%), *Sphingomonas* (purple, FeS_2_ = 6%), *Methylotenera* (brown, FeS_2_ = 5%), and *Sphingopyxis* (yellow, FeS_2_ = 4%). The remainder (gray) represents genera that each comprise less than 4% of the total population. **(B)** Distribution and composition of sulfur (S) in cross sections obtained from the incubated metacinnabar. Left: PCA analysis of the seven energy S K-edge maps indicate that three components account for the S speciation whose XANES spectra match unreacted metacinnabar (*a* = β-HgS), elemental sulfur (*b* = S^0^), and sulfate (*c* = SO^2−^_4_). Right: μ-XRF map illustrating the presence of the oxidation product S^0^ (red) and isolated spots of sulfate (green) on the HgS surface (blue) (spot size = 5 μm). Scale bar = 60 μm.

We first characterized the microbial colonization and mineralogical transformations of metacinnabar mineral sections that were emplaced below the sediment-water interface (2.5–5 cm) within the aerobic hyporheic zone of a Hg-contaminated creek. Culture-independent analysis of the microbial communities showed that the metacinnabar surfaces were extensively colonized by chemosynthetic bacteria, which accounted for a striking majority (>92%) of the taxonomically classified microbial community (Figure 1A, Supplementary Table S2). This chemosynthetic population was composed predominantly of members of the genera *Thiobacillus* (71%), *Sulfuricurvum* (13%), and *Sulfuricella* (8%), genera that have the demonstrated ability to oxidize reduced sulfur compounds. Pyrite surfaces similarly enriched for these organisms, but to a lower relative magnitude (Figure 1A). The metacinnabar surface evidently enriched for these sulfur-oxidizing organisms as previous characterization of these creek sediments revealed a microbial community that was phylogenetically diverse and taxonomically distinct from those found on the mineral incubations (Vishnivetskaya et al., 2011). Furthermore, the *Thiobacillus* population on the metacinnabar surface was mainly (>50%) comprised of a single species, *T. thioparus*. *T. thioparus* is a common neutrophilic obligate aerobic sulfur-oxidizing bacterium that couples the oxidation of various sulfur species (e.g., sulfur, thiosulfate, sulfide) to the reduction of oxygen (Kelly and Wood, 2000). This organism (a beta-proteobacterium) is distinct from the iron- and sulfur-oxidizing *Acidithiobacillus* species (gamma-proteobacteria) that have previously demonstrated metacinnabar dissolution at acidic pH. Specifically, *T. thioparus* lives solely in circumneutral environments and cannot respire or otherwise oxidize Fe(II). Thus, the proliferation of these organisms on the metacinnabar surface may have ensued due to enhanced access to mineral-hosted sulfur to fuel respiration.

In fact, metacinnabar oxidation is implicated by observations of sulfide oxidation products on the surface of cross sections of the same metacinnabar incubated in the creek sediments (Figure 1B). Specifically, principal component analysis (PCA) of X-ray fluorescence maps (XRF) were collected at seven different energies around the sulfur K-edge that are indicative of a full range of organic and inorganic sulfur species. This analysis indicated that three unique components were in the incubated metacinnabar samples. These components were identified as HgS, elemental sulfur (S^0^), and sulfate (SO_4_) by obtaining spot X-ray absorption near-edge structure (XANES) spectra and comparing them to model compounds (Figure 1B). Using unique spectral signatures for these phases, multiple-energy XRF maps for these three spectral components revealed heterogeneous surface rinds on the incubated metacinnabar surface composed primarily of elemental sulfur with isolated sulfate regions (Figure 1B). Oxidized sulfur products on the surface of metacinnabar incubated in sterile freshwater were not observed (Supplementary Figure S1).

**Figure 2:**
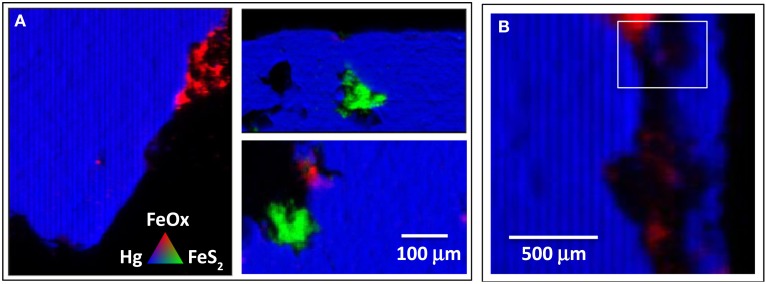
**Distribution and composition of Fe and Hg in cross sections obtained from field-incubated metacinnabar**. Multiple energy maps were collected on beamline 2-3 at the Stanford Synchrotron Radiation Laboratory (see Methods). PCA analysis of the five energy Fe K-edge maps indicate that only two components account for the Fe speciation. Based on comparison to Fe XANES standard spectra, these components are a ferrihydrite-like Fe (oxy)hydroxide (FeOx) and pyrite (FeS_2_). **(A)** These μ-XRF maps illustrate that Fe within the metacinnabar mineral is below detection except for regions containing pyritic inclusions (green). Significant oxidation of regions near the pyritic inclusions is not observed, however iron (oxy)hydroxide precipitates are observed in discrete regions on the metacinnabar surface (as would be expected if small amount of Fe were released from metacinnabar to the aerobic porewater). **(B)** An expanded image of the boxed region shown in Figure 1, shows some regions of oxidized iron within the map showing extensive elemental sulfur rinds (Figure 1). However, the oxidized iron phases are limited to the upper area of the map (spot size = 5 μm).

Further, energy specific XRF maps reveal the presence of oxidized iron, as Fe(III) (hydr)oxides, in isolated regions on the surface of metacinnabar incubated in the creek sediments (Figure 2). Iron is present in the metacinnabar primarily as pyritic inclusions (Figure 2) and likely also as a trace co-precipitate within the structure. These results indicate that the metacinnabar surface was in fact dissolving leading to the formation of substantial surficial S^0^ rinds and Fe (hydr)oxide precipitates. This oxidation may be a direct or indirect consequence of the activity of the chemosynthetic bacterial communities that had extensively colonized the mineral surface. In particular, these elemental sulfur rinds coupled with the extensive colonization of the surface by the obligate sulfur-oxidizer *T. thioparus*, an organism that cannot oxidize Fe(II), point to a role for microbially-mediated sulfur oxidation in the oxidative dissolution of metacinnabar in these incubations.

### Metacinnabar dissolution by *Thiobacillus* cultures

**Figure 3:**
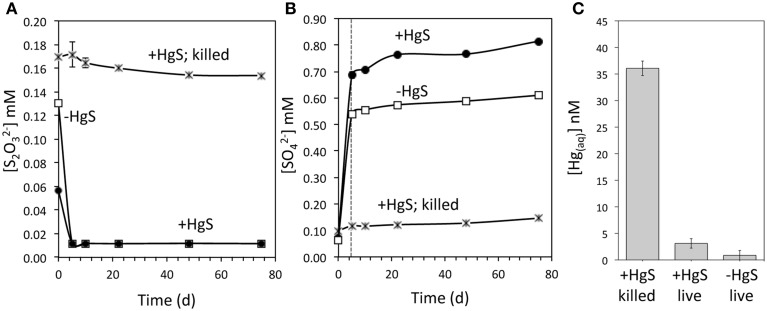
**Aqueous sulfur and mercury dynamics in** ***Thiobacillus*** **incubations with metacinnabar**. Aqueous thiosulfate **(A)**, sulfate **(B)**, and Hg **(C)** concentrations within *Thiobacillus thioparus* incubations initiated with ~100 μM thiosulfate and metacinnabar. Thiosulfate concentrations are below detection following 5 days of reaction **(A)** corresponding with an increase in the oxidation product sulfate **(B)**. Dashed vertical line in **(B)** indicates time point following which thiosulfate is no longer detected. In the absence of metacinnabar, sulfate production in excess of the stoichiometric amount of sulfate produced from thiosulfate oxidation (2 moles of sulfate produced per mole of thiosulfate), originates from the oxidation of elemental sulfur introduced from the inoculum. Minimal abiotic thiosulfate oxidation and sulfate production are observed in killed controls. A background sulfate level of less than 10 μM is observed accounting for any sulfate carryover from the killed inoculum as well as abiotic oxidative dissolution and release of surface sorbed sulfate (see Table 1). In contrast to killed incubations, aqueous Hg concentrations **(C)** in the presence of live *Thiobacillus* cells after 10 days of reaction are significantly lower. Error bars for most time points are smaller than the symbol. Error bars are the standard deviation for method duplicates. In a parallel set of incubations with metacinnabar and the same medium but no cells, aqueous Hg levels are 34 nM after 8 days (similar to 37 nM for killed controls). Further, sulfate production/release in these cell free controls within 10 days is 0.7 μM d^−1^, again similar to the killed controls (1.7 μM d^−1^).

Incubation of *T. thioparus* axenic cultures with metacinnabar at varying thiosulfate concentrations further points to microbially induced metacinnabar oxidation. In incubations containing an environmentally relevant thiosulfate concentration (initially ~60–100 μM), thiosulfate was consumed by *T. thioparus* within the first 5 days (Figure 3A) with the dominant oxidation product being sulfate (Figure 3B), formed through a S^0^ intermediate (Supplementary Figure S2) as shown previously (Sattley and Madigan, 2006). Following thiosulfate consumption, sulfate concentrations in the presence of metacinnabar continued to increase at a faster rate than in its absence (17 and 10 μM day^−1^ sulfate production, respectively, Figure 3B). Further, a substantially higher sulfate concentration was observed in the presence of metacinnabar relative to its absence (Figure 3B) and was formed in excess of that supported solely by oxidation of added thiosulfate and residual elemental sulfur in the inoculum (see Table 1). Together, these results suggest that metacinnabar is in fact fueling sulfur respiration and thereby undergoing microbially mediated oxidative dissolution.

**Table 1:** **Microbial contribution to metacinnabar dissolution and microbially mediated dissolution rates**.

**Initial Thiosulfate Concentration**	**x days**	***H_x_* μM**	***N_x_* μM**	***A_x_* μM**	***C*_0_ μM**	***M_x_* μM**	**Contribution of microbial processes to total HgS dissolution (%)**	***R* μMol (sulfate) m^−2^ d^−1^**
0 μM	11	1024	927	57	7	47	48	16.2
	10	710	560	120	60	90	60	34.1
60 μM	22	760	570	120	60	130	68	22.4
	75	810	610	150	60	110	55	–

*The microbial contribution to metacinnabar dissolution observed in our incubations was calculated using sulfate production as a proxy for metacinnabar dissolution as has been reported previously (Barnett et al., 2001; Holley et al., 2007) and calculated using the equation, M_x_ = (H_x_ – N_x_) – (A_x_ – C_0_), where M_x_ is the microbial contribution to sulfate production x days after inoculation, H_x_ is the sulfate produced in live Thiobacillus incubations in the presence of metacinnabar (+HgS), N_x_ is sulfate produced in live Thiobacillus incubations in the absence of metacinnabar (–HgS), A_x_ is sulfate produced in killed cell incubations in the presence of metacinnabar (+HgS), and C_0_ is the initial sulfate concentration (t = 0) in live Thiobacillus incubations in the absence of metacinnabar (–HgS). The term N_x_ represents sulfate production attributed to thiosulfate and elemental sulfur oxidation, as well as carryover sulfate from the inoculum, and (H_x_ – N_x_) represents sulfate release from metacinnabar from biotic and abiotic processes. The term (A_x_ – C_0_) represents abiotic oxidative dissolution and release of any sulfate bound to the metacinnabar surface, with C_0_ representing carryover sulfate from the inoculum. M_x_, H_x_, N_x_, A_x_, and C_0_ are reported in μM. Microbially mediated metacinnabar dissolution rates (R) are calculated by taking into account mineral loading (2 g metacinnabar L^−1^ incubation) and surface area (0.1321 m^2^ g^−1^) and are presented as μMoles (sulfate) m^−2^ d^−1^*.

**Figure 4:**
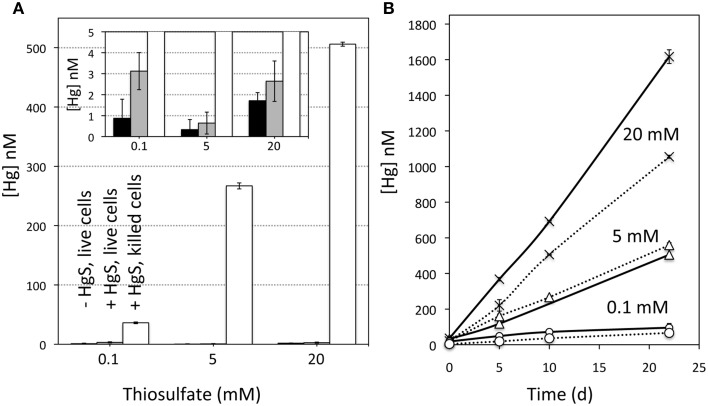
**Aqueous Hg concentrations in incubations. (A)** Aqueous Hg concentrations (nM) in incubations containing various initial thiosulfate concentrations after 10 days of reaction. The conditions included (black bars) live cells, no metacinnabar; (gray bars) live cells plus metacinnabar, and (white bars) killed cells plus metacinnabar. The inset shows data up to 5 nM Hg to illustrate the differences in the live incubations that are masked in the full range due to the high Hg in killed cell incubations. **(B)** Aqueous Hg concentrations (nM) over time as a function of thiosulfate concentration (0.1, 5, and 20 mM) within incubations containing killed cells and metacinnabar. The solid lines were conducted in a basal freshwater medium containing EDTA, while the dashed lines contained no EDTA. The standard deviation of two method replicates is indicated.

**Figure 5:**
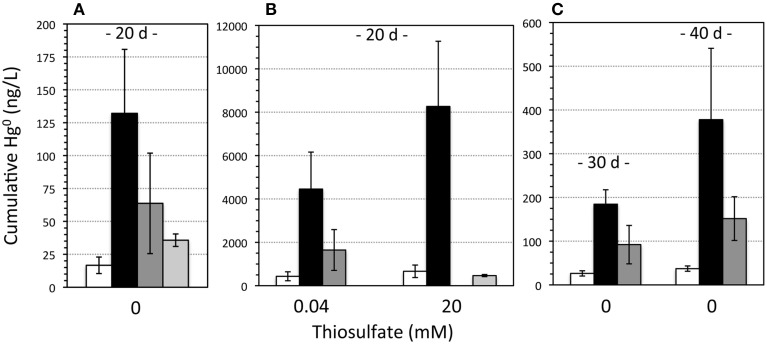
**Gaseous mercury (Hg^0^) measured in** ***Thiobacillus*** **incubations**. Concentrations are presented as ng Hg^0^ per liter of culture volume. **(A,B)** Gaseous Hg production after 20 days within incubations containing no HgS (white bars), HgS plus live *Thiobacillus* (black bars), HgS plus live *Thiobacillus* at a lower cell density (~10× fewer cells) (dark gray bars), and HgS plus killed *Thiobacillus* cells (light gray bars) and with an initial thiosulfate concentration of **(A)** zero and **(B)** 0.04 or 20 mM thiosulfate. **(C)** Gaseous Hg produced in the zero thiosulfate incubations after 30 and 40 days. Error bars are the standard deviation for two to four biological replicates.

Yet, despite apparent HgS dissolution, negligible levels of aqueous Hg were detected. Dissolved Hg^2+^ was observed in incubations containing killed (formaldehyde-treated or autoclaved) *T. thioparus* cells, with concentrations ranging from 36 nM to more than 500 nM within 10 days (Figures 3C, 4). In contrast, aqueous Hg was below 3 nM in equivalent incubations containing viable cells (Figures 3C, 4) and even after extended periods of time (>11 weeks). The explanation for this is that the aqueous Hg released during metacinnabar dissolution was rapidly volatilized to gaseous Hg^0^ in the presence of viable *T. thioparus*. In fact, a substantial amount of gaseous Hg^0^ was observed in metacinnabar incubations with viable cells (Figure 5). The cumulative gaseous Hg^0^ released in the presence of *T. thioparus* varied with reaction time, cell density and initial thiosulfate concentration and ranged from ~100 to >8000 ng Hg^0^ per liter of culture over a 20-day period (Figures 5A,B).

### Impact of thiosulfate on metacinnabar dissolution

In incubations with initial thiosulfate concentrations of 0 and 60 μM, 48–68% of the excess sulfate production, could be solely attributed to microbial-enhanced HgS dissolution (see Table 1 and Supplementary Figure S3). Even in the absence of any exogenous thiosulfate, Hg^0^ formation was still occurring after 40 days amounting to nearly 400 ng of Hg(0) per liter of culture (Figure 5C) and further hinting at sustained microbial respiration and oxidation of metacinnabar-derived sulfur. Meanwhile, minimal volatilization was observed in incubations with killed cells, and with viable cells in the absence of metacinnabar (i.e., background Hg levels in air). The non-metabolic contribution in the killed cell controls is likely due to abiotic Hg(II) reduction by Fe(II) (Wiatrowski et al., 2009; Amirbahman et al., 2013) impurities within the metacinnabar structure (Figure 2). Elevated Hg^0^ production in the presence of live cells demonstrates that Hg was in fact released from metacinnabar and then volatilized to Hg^0^ by *T. thioparus*. Many sulfur-oxidizing organisms possess the enzyme mercuric reductase (Benson et al., 2009), *merA*, which reduces Hg^2+^ to Hg^0^ (Barkay et al., 2003). Although *T. thioparus* may also have other strategies or proteins capable of Hg^2+^ reduction, PCR amplification using *mer*-specific primers and sequencing confirmed that *T. thioparus* possesses the *merA* gene. BLAST analysis of the *merA* fragment nucleotide sequence revealed a 98% sequence similarity to *T. thioparus* DSM 505 and 85% similarity to *Thiobacillus denitrificans* ATCC 25259, both containing putative mercuric reductase genes. Supplementary Data S1 includes the 285-bp sequence for the *merA* fragment identified. Hence, the *T. thioparus* strain used in these investigations has the genetic potential to reduce Hg^2+^ as observed in our incubations here.

Addition of higher thiosulfate levels led to enhanced metacinnabar dissolution and volatilization. In the absence of microbial activity (e.g., killed cells), aqueous Hg^2+^ levels increased with increasing initial thiosulfate concentration (Figure 4), indicating that thiosulfate stimulated metacinnabar dissolution. Thiosulfate is a strong Hg^2+^ complexing ligand (Crespo-Medina et al., 2009) and MINTEQ modeling indicated that under the experimental conditions tested, nearly all aqueous Hg^2+^ was complexed to thiosulfate, predominantly as Hg(S_2_O_3_)^−2^_2_ (98%) under low (100 μM) thiosulfate conditions and Hg(S_2_O_3_)^−4^_3_ (77%) under high (20 mM) thiosulfate concentrations. In the presence of live *T. thioparus*, higher thiosulfate concentrations also led to higher cell densities (Supplementary Figure S4) ultimately resulting in a greater mass of Hg volatilized over a given time period (Figure 5). The Hg volatilization rate (~100–500 ng L^−1^ d^−1^) after 11–30 days of incubation, when most thiosulfate had been consumed by cells (concentration remaining 0–5 μM), however is similar when normalized to cell abundance (~50 ag Hg cell^−1^ d^−1^) for a wide range of initial thiosulfate concentrations (40 μM to 20 mM) (Supplementary Figure S5). Thus, thiosulfate had a dual effect on HgS dissolution, by acting as both a complexing ligand inducing abiotic metacinnabar dissolution before it was consumed through cell respiration and by thereby supporting higher cell densities to stimulate microbially induced dissolution. Our findings demonstrate that at environmentally relevant concentrations, even a small increase in initial thiosulfate abundance (from <4 to 40 μM) resulted in a large enhancement of Hg volatilization (~35 times, Figure 5).

**Figure 6:**
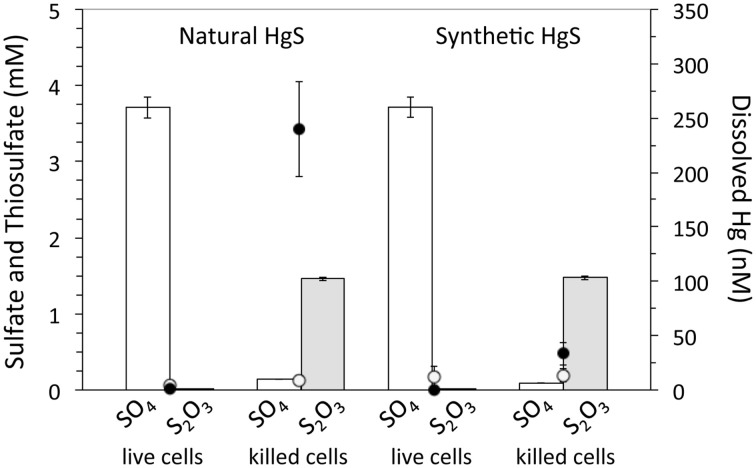
**Sulfate, thiosulfate, and dissolved Hg within** ***T. thioparus*** **incubations in thiosulfate spike experiments**. Sulfate (white bars) and thiosulfate (gray bars) concentrations in live and killed cell incubations 12 days after inoculation and following daily 100 μM additions of thiosulfate. Dissolved Hg measured in incubations 15 h (open circles) and 12 days (solid circles) after inoculation. Error bars indicate the standard deviation of three replicate incubations.

**Figure 7:**
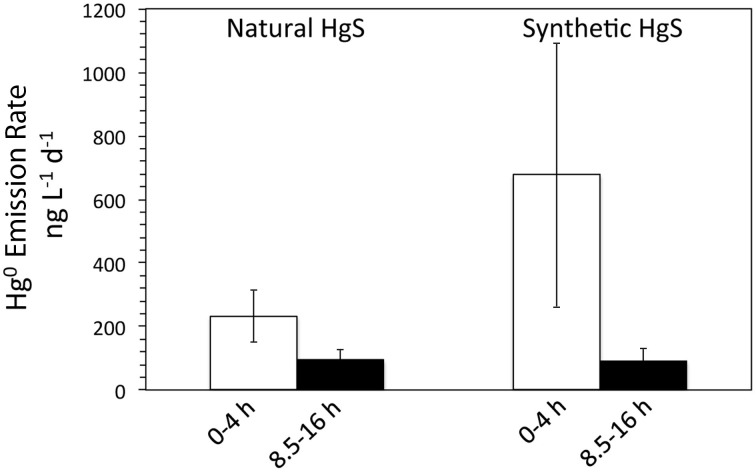
**Gaseous Hg emission rates within** ***T. thioparus*** **incubations in thiosulfate spike experiments**. Average gaseous Hg emission rates within live *T. thioparus* incubations with natural and synthetic metacinnabar and a daily 100 μM thiosulfate spike. Each Hg^0^ emission rate displayed represents an average of 5 measurements (*n* = 5) that were taken on 5 different days (days 4–9 during incubation) and grouped for timepoints obtained either 0–4 or 8.5–16 h following the spike. Error bars indicate the standard deviation of measurements taken from three replicate incubations and the five measurements for each time sampling. The high variability in the incubations is due to deviations in the metabolic activity of *Thiobacillus* in the biological replicates and likely also changes in mineral activity/reactivity over the course of the experiment.

Thiosulfate is a major intermediate sulfur species in environmental systems formed through both oxidative and reductive legs of the sulfur cycle, leading to high thiosulfate fluxes despite variable steady-state concentrations (typically low- to mid-micromolar concentrations, but as high as 3 mM in estuarine and marine waters and sediments) (Luther et al., 1986; Jorgensen, 1990a,b; Zopfi et al., 2004; Mullaugh et al., 2008). Accordingly, a subset of experiments was performed to determine the impact of sustained fluxes of thiosulfate on metacinnabar dissolution and Hg volatilization, as well as to compare natural metacinnabar (surface area = 0.1321 ± 0.0007 m^2^ g^−1^) to a synthetic iron-free, finer grained, and higher surface area metacinnabar phase (surface area = 1.2804 ± 0.0045 m^2^ g^−1^). Daily 100 μM thiosulfate spikes were quickly oxidized within hours in the presence of live *T. thioparus* with concomitant sulfate production, while thiosulfate was minimally consumed in killed incubations (Figure 6). Dissolved Hg in killed incubations increased over the course of the experiment, due to thiosulfate-induced Hg complexation and dissolution, with a more pronounced increase in natural than in synthetic metacinnabar incubations (Figure 6). Dissolved Hg concentrations in natural metacinnabar incubations were ~7× higher than with synthetic metacinnabar, which may be a consequence of greater Hg adsorption to the higher surface area synthetic phase. Equivalent dissolved Hg levels were obtained for solutions filtered with 0.02 and 0.2 μm filters, indicating that the released Hg from metacinnabar was in fact dissolved Hg and there was no significant nanoparticulate contribution. As expected, gaseous Hg(0) was observed in both the natural and synthetic HgS incubations; albeit with a high degree of variability likely due to an offset in the microbial activity of the biological replicates (Figure 7). Overall, a general trend toward decreasing Hg volatilization rates was observed over time following the thiosulfate spike (Figure 7).

The timing of thiosulfate additions had a substantial effect on the dynamics of microbially-induced volatilization of mineral-hosted Hg, with release of volatile Hg from natural and synthetic metacinnabar occurring rapidly within the first few hours following the thiosulfate spike, and decreasing volatilization after thiosulfate is consumed by *T. thioparus* (Figure 7). Thus, in natural systems where a low continuous flux of thiosulfate is likely present, microbial volatilization of Hg-thiosulfate complexes may lead to substantial metacinnabar dissolution and represent a significant source of gaseous Hg(0). Further, despite synthetic metacinnabar having a higher surface area and thus theoretically higher solubility, enhanced abiotic dissolution of natural metacinnabar here points to more complex controls on metacinnabar dissolution and likely highlights the importance of impurities such as iron.

## Conclusion

Here we show that authigenic HgS is not merely a sink for Hg within non-acidic natural environments and instead is a source of gaseous Hg (Figures 5, 7). Volatilization of metacinnabar-hosted Hg is a coupled process involving metacinnabar dissolution (Figure 4) and microbial reduction of released Hg (Figures 5, 7). Both microbial activity and thiosulfate enhance Hg release through presumably oxidative and ligand-promoted dissolution processes, respectively. Surprisingly, it appears that metacinnabar could serve as a respiratory source of sulfur in the absence of thiosulfate for *T. thioparus* (Figure 3, Table 1) and likely other sulfur-oxidizing bacteria (e.g., *Sulfuricurvum* and *Sulfuricella*) explaining their extensive colonization on metacinnabar in sediments (Figure 1). Utilizing HgS as a metabolic substrate, however, likely limits the sulfur-oxidizing community to those that can also detoxify the Hg released upon dissolution. Metacinnabar dissolution by thiosulfate (Figure 4) or other Hg complexing ligands will also provide sulfide or other S intermediates (e.g., formed via abiotic oxidation of sulfide) further fueling the metabolism of sulfur-oxidizing organisms. Within aerobic systems, as explored here, this volatilized Hg may evade to the atmosphere. Under fluctuating redox conditions, however, methylation may compete for the released Hg from metacinnabar. The ultimate fate of released Hg from metacinnabar will undoubtedly be a function of the system geochemistry and resident microbial community. Regardless of the dissolution mechanisms and fate of Hg, these complex dynamics challenge the notion that metacinnabar serves as a static Hg host within non-acidic sediments.

These findings point to HgS as an underappreciated source of Hg to the environment. Based on these Hg volatilization rates, this process could account for a significant amount of Hg release from sediments and soils into the atmosphere. The microbially enhanced dissolution rates observed here, excluding any abiotic contribution, are 9–1000 times higher than known oxidative dissolution rates (3.15 × 10^−2^ to 1.90 μmol (sulfate) m^−2^ d^−1^) (Barnett et al., 2001; Holley et al., 2007) (see Table 1). A reasonable extrapolation for the environmentally relevant Hg volatilization rate from the processes observed in this study is 34 ng Hg m^−2^ h^−1^ (see Table 1), which falls within the range of observed emissions from mineral mercury enriched areas (2–440 ng Hg m^−2^ h^−1^) (Gustin, 2011). Our findings may provide a mechanistic understanding, at least in part, for these field observations. Further, as a first approximation, extrapolating this area normalized rate to an area roughly equivalent to that of all wetlands, redox dynamic systems rich in metal sulfides and thiosulfate, yields a Hg release rate of 1 Mmoles yr^−1^ (see Supplementary Table S3). For scale, total Hg emissions from terrestrial soils are estimated at 15 Mmoles yr^−1^ (Smith-Downey et al., 2010) with the contribution from geologically enriched soils estimated at 2.5–7.5 M yr^−1^ (Gustin, 2011). With that being said, the HgS loadings used in this study were employed to mimic Hg contaminated systems that are common worldwide (Barnett et al., 1997), and thus the application of this process to regional and global scale budgets that take into account also lower HgS environments requires further exploration and validation.

## Author contributions

AV and CH jointly conceived of the study and designed the experiments. AV performed the experiments; AV, TZ, and CH collected the data; CS, SW, and CL assisted in data analysis; SB assisted with fieldwork. AV and CH wrote the manuscript with input from TZ, CL, CS, SW, and SB.

### Conflict of interest statement

The authors declare that the research was conducted in the absence of any commercial or financial relationships that could be construed as a potential conflict of interest.
