# Supplementary material for: Microbial- and thiosulfate-mediated dissolution of mercury sulfide minerals and transformation to gaseous mercury
Source: Front Microbiol. 2015 Jun 23;6:596. doi: 10.3389/fmicb.2015.00596 (PMC4477176; doi:10.3389/fmicb.2015.00596)
Supplement: Supplementary file 1 [file SupplementaryMaterial.PDF]

Supplementary Material

Microbial- and Thiosulfate-Mediated Dissolution of Mercury Sulfide Minerals and Transformation to Gaseous Mercury

Adiari I. Vázquez-Rodríguez[1], Colleen M. Hansel[2], Tong Zhang[2], Carl H. Lamborg[2], Cara M. Santelli[3], Samuel M. Webb[4], Scott C. Brooks[5]

[1]School of Engineering and Applied Sciences, Harvard University, Cambridge, MA, USA
[2]Department of Marine Chemistry and Geochemistry, Woods Hole Oceanographic Institution, Woods Hole, MA, USA
[3]Smithsonian Institution, National Museum of Natural History, Department of Mineral Sciences, Washington, DC, USA
[4]Stanford Synchrotron Radiation Lightsource, Menlo Park, CA, USA
[5]Environmental Sciences Division, Oak Ridge National Laboratory, Oak Ridge, TN, USA

*** Correspondence:** Colleen Hansel, Woods Hole Oceanographic Institution, 266 Woods Hole Road, MS52, Woods Hole, MA 02543 USA, 508-289-3738, chansel@whoi.edu

Supplementary Materials

Supplementary Table S1. Chemical and physical parameters of the East Fork Poplar Creek channel pore waters at EFK 22 October 2010. ND denotes values below instrument detection limit. Uncertainty in the measurement is reported as ± 1 standard error.

Parameter	Depth (cm)			
	0.0	7.6	15.2	22.9
Temperature (°C)	14.7	16.5	16.1	16.7
pH	8.0	8.1	8.0	7.8
Conductivity (μS/cm)	354	356	355	316
ORP (mV)	85	69	118	115
Element Concentration (mg/L)				
Ca	41.5 ± 1.2	39.9 ± 1.2	40.7 ± 1.2	32.1 ± 1.2
Mg	12.9 ± 1.1	12.4 ± 1.1	12.6 ± 1.1	14.7 ± 1.1
Na	9.7 ± 1.1	9.2 ± 1.1	9.1 ± 1.1	5.7 ± 1.1
Dissolved Hg (ng/L)	19.05 ± 4.8	12.8 ± 0.4	12.1 ± 0.4	7.1 ± 0.4
Ion Concentration				
NH_4^+ (mg/L)	82.7 ± 0.8	26.8 ± 0.7	5.9 ± 0.8	15.9 ± 0.7
SRP (mg/L)	26.9 ± 0.5	37.4 ± 0.5	46.9 ± 0.5	30.4 ± 0.5
Sulfide (mg/L)	ND	ND	ND	ND
Cl^- (mg/L)	10.6 ± 0.1	10.7 ± 0.1	10.6 ± 0.1	4.2 ± 0.1
Br^- (mg/L)	ND	ND	ND	ND
NO_3^- (mg/L)	7.3 ± 0.1	7.7 ± 0.1	7.7 ± 0.1	3.3 ± 0.1
SO_4^{2-} (mg/L)	36.8 ± 0.1	36.4 ± 0.1	36.3 ± 0.1	31.2 ± 0.1

Supplementary Table S2. Taxonomic bacterial composition of incubated metacinnabar and pyrite sections. Genus-level classification of bacterial sequences obtained from metacinnabar and pyrite surfaces incubated within the East Fork Poplar Creek 2.5 to 5 cm below the sediment-water interface. Percentages represent the number of sequences assigned to each genus divided by the total number of classified sequences. Only genera that comprise over 4% of the classified population are shown.

**Metacinnabar**			**Pyrite**	
Thiobacillus	71%		Thiobacillus	26%
Sulfuricurvum	13%		Hyphomicrobium	17%
Sulfuricella	8%		Sulfuricurvum	6%
Methyloversatilis	4%		Sphingomonas	6%
			Methyloversatilis	5%
			Methylotenera	5%
			Sphingopyxis	4%

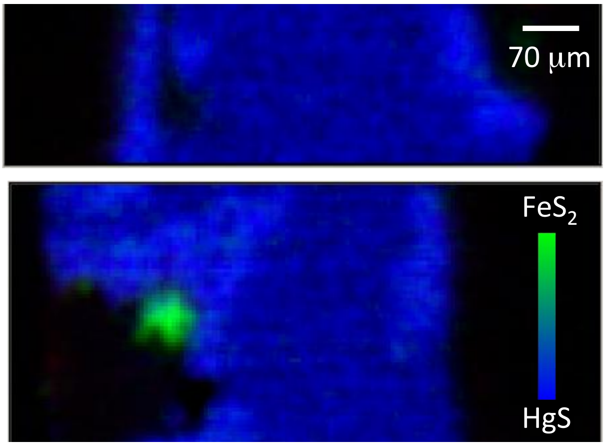

Supplementary Fig. S1. Distribution and composition of sulfur (S) in cross sections obtained from control metacinnabar mineral slabs incubated in sterile freshwater for 4 weeks. As in Figure 1, PCA analysis was conducted at the 7 energy S K-edge maps to indicate the number of components that account for S speciation. Only two components were identified for the control sections, whose XANES spectra match unreacted metacinnabar (blue = β-HgS) and another metal sulfide phase whose XANES spectra most closely matches pyrite (green = FeS_2) (spot size = 5 μm).

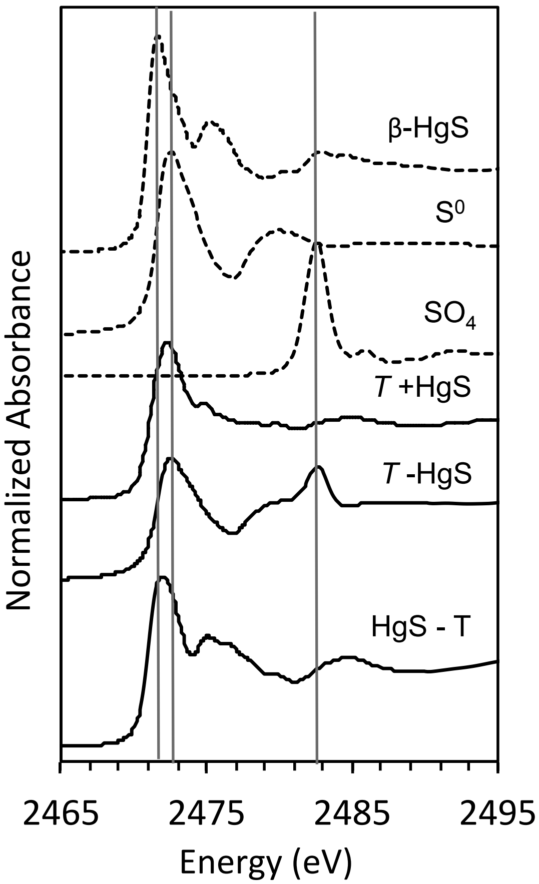

Supplementary Fig. S2. S K-edge XANES spectra of bulk sulfur speciation within *Thiobacillus* (T) incubations either in the presence or absence of metacinnabar after one week of incubation in the same incubation medium described in the methods. In the absence of metacinnabar (T –HgS), the dominant S speciation is elemental sulfur (S^0) and sulfate (SO_4), illustrating these two species as the byproduct of thiosulfate oxidation by *Thiobacillus*. For incubations with metacinnabar, the spectra is difficult to deconstruct, however, in the presence of the high sulfide signature in the spectra. Nevertheless, a subtle shift in the white line energy to higher oxidation states coincide with the presence of elemental S in these incubations as well (T +HgS). In contrast, for metacinnabar incubated within the medium in the absence of *Thiobacillus* (HgS – T), a broadening of the peak is observed but not as substantial a shift in the white line suggesting negligible oxidation. These results suggest that elemental S is an intermediate in sulfide oxidation to sulfate, similar to that of thiosulfate oxidation. Standard spectra are also provided for reference (dotted lines).

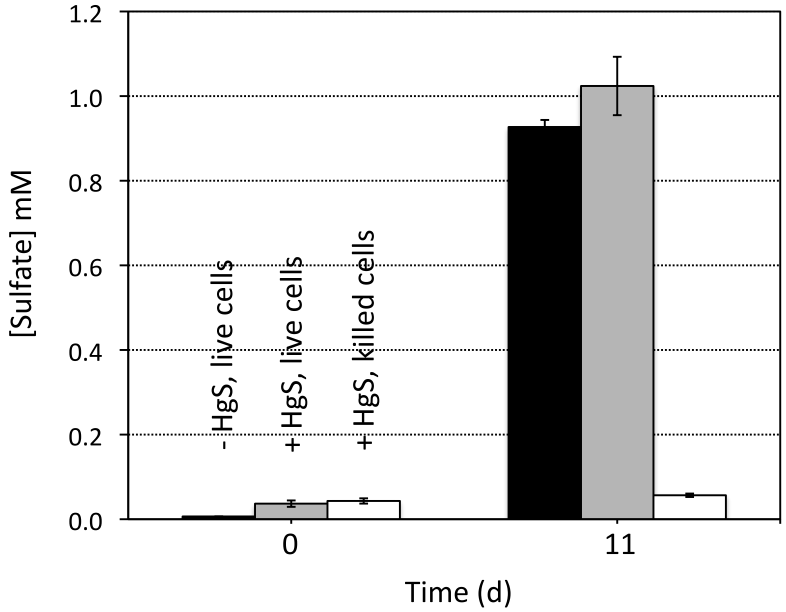

Supplementary Fig. S3. Aqueous sulfate concentrations within *Thiobacillus* incubations initiated with zero added thiosulfate. In all cultures, elemental S is present within the inoculum, with the concentration equivalent between all samples. In the absence of metacinnabar (-HgS), sulfate production is formed solely via oxidation of this transferred S(0). In the presence of metacinnabar (+HgS), the sulfate produced via oxidation of metacinnabar-derived sulfide is that in excess of that observed in the absence of metacinnabar (~100 μM) and that observed in the presence of metacinnabar (+HgS) and dead cells (~50 μM) (see Table 1).

Supplementary Materials

Supplementary Data S1. *Thiobacillus thioparus* ATCC 8158 *merA* gene fragment sequence. This 285 bp gene sequence at the 3′ end of *merA* was amplified using PCR primers A1s-n.F and A5-n.R (Chadhain et al., 2006).

>merA_ATCC8158

ACCATCGTCAGGTAGGGGAACAACTGCCCCGCCAGATCCGCGACAGTCATGCGGTT
CTTTATCGCCATCGCGGCGGTCTGGATGATCTCGCCACCTTCTCCCGCCAGCACCTG
CGCACCCAGCAGGCGTCCGGTGTCTCTGTCCGCCACGAGTTTGATGAAGCCGCGGGT
ATCGAAGTTGGCCAGTGCGCGCGGCACGTTGTCCAGCGTCAAGGTGCGGCTGTCGGT
TGCCATGCCTAGCCTGCTTGCCTGCGCTTCGGTGAGACCGACCGTCGCCACTTGCGG
A

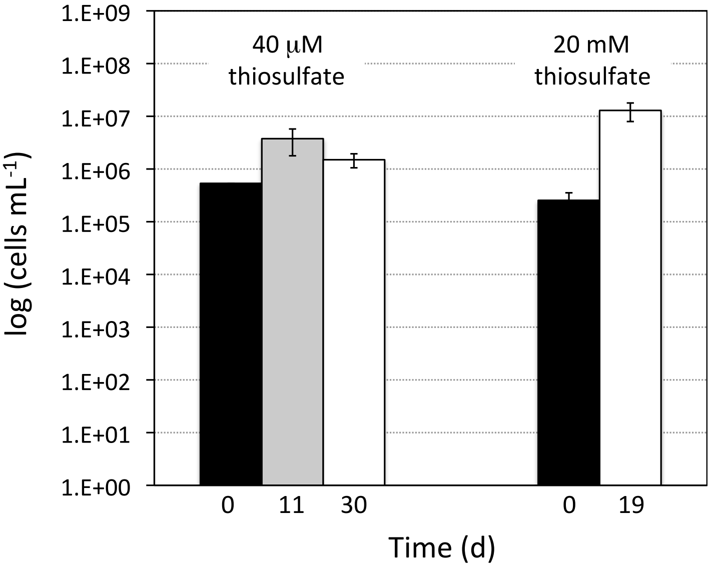

Supplementary Fig. S4. Bacterial cell counts (cells mL^{-1}) in incubations within the initial inoculum (t=0) and over time, illustrating growth under both conditions and higher cell counts obtained in the presence of higher initial thiosulfate concentrations.

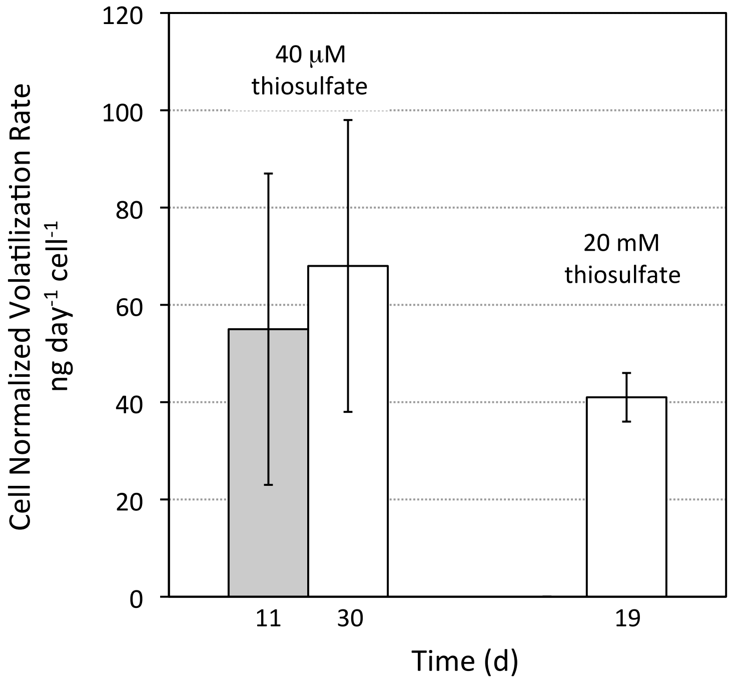

Supplementary Fig. S5. Cell normalized Hg volatilization rates within *Thiobacillus* incubations (+HgS) initiated with 40 μM and 20 mM thiosulfate. Gaseous Hg emission rates collected on the day indicated are divided by the number of cells present at that timepoint and presented as ng Hg^0 per day per cell. Most of the initial thiosulfate had been consumed at these timepoints (concentration remaining 0 – 5 μM), and the cell normalized Hg volatilization rate is similar for initial thiosulfate concentrations shown here.

Supplementary Table S3. We extrapolated Hg volatilization rates from metacinnabar assuming bacterial cell density for Hg-contaminated saturated soils (Vishnivetskaya *et al.* 2011), and a typical saturated soil density of 1.5 g/cm^3. We assume a percentage of the bacterial community is mercury resistant based on resistance observed in Hg-contaminated environments (Crespo-Medina *et al.* 2009). We assume all resistance to result in Hg-volatilization. To constrain our estimate, we take only the top 2 cm of soil into account, where emitted Hg^0 might be expected to diffuse into the atmosphere (Mazur et al. 2013) and conditions might be expected to be aerobic.

Three extrapolations were performed: 1) a lower bound, 2) an upper bound, and 3) a reasonable estimate taking into account low end, high end, and a reasonable estimation of each assumed parameter, respectively. Three cell normalized Hg-volatilization rates were used as observed in our study within lab incubations after (a) 19 days with an initial thiosulfate concentration of 20 mM (41 ag $cell^{-1}$ d^{-1}), (b) 11 days with an initial thiosulfate concentration of 40 mM (55 ag $cell^{-1}$ d^{-1}), and (c) 30 days with an initial thiosulfate concentration of 40 mM (68 ag $cell^{-1}$ d^{-1}). This exercise results in a *Projected Mercury Release Rate* (mmoles Hg m^{-2} yr^{-1}).

Using the "reasonable estimates", we further show predicted global emissions for the extrapolated land area normalized volatilization rate. Here we take into account the global wetland area only, which is projected as 6.3 x 10^{11} m^2 (Schuyt and Brander, 2004).

Conditions	Lower Bound	Upper Bound	Reasonable Estimate
For cell-normalized mercury volatilization rate of 41 ag $cell^{-1}$ day^{-1}			
Soil Cell Density (cells/g)[a]	6.75E+07	3.62E+09	1.00E+08
Hg resistance (% of total bacterial population)[b]	0.20%	24.60%	1%
Projected Mercury Release Rate (umoles Hg m^{-2} yr^{-1})[c]	*0.30*	*1993*	***2.2***
Projected Global Mercury Release Rate (Mmoles Hg yr^{-1})[d]			***1.4***
For cell-normalized mercury volatilization rate of 55 ag $cell^{-1}$ day^{-1}			
Soil Cell Density (cells/g)[a]	6.75E+07	3.62E+09	1.00E+08
Hg resistance (% of total bacterial population)[b]	0.20%	24.60%	1%
Projected Mercury Release Rate (umoles Hg m^{-2} yr^{-1})[c]	*0.41*	*2674*	***3.0***
Projected Global Mercury Release Rate (Mmoles Hg yr^{-1})[d]			***1.9***
For cell-normalized mercury volatilization rate of 68 ag $cell^{-1}$ day^{-1}			
Soil Cell Density (cells g-1)[a]	6.75E+07	3.62E+09	1.00E+08
Hg resistance (% of total bacterial population)[b]	0.20%	24.60%	1%
Projected Mercury Release Rate (umoles Hg m^{-2} yr^{-1})[c]	*0.50*	*3306*	***3.7***
Projected Global Mercury Release Rate (Mmoles Hg yr^{-1})[d]			***2.3***

[a]Vishnivetskaya et al., 2011

[b]Crespo-Medina et al., 2009

[c]projected for top 2 cm of soil

[d]projected including a global wetland area of 6.3x10^{11} m^2

Supplemental Materials References

Crespo-Medina, M., Chatziefthimiou, A.D., Bloom, N.S., Luther, G.W., Wright, D.D., Reinfelder, J.R., Vetriani, C., and Barkay, T. (2009). Adaptation of chemosynthetic microorganisms to elevated mercury concentrations in deep-sea hydrothermal vents. *Limnology and Oceanography* 54**,** 41-49. doi: 10.4319/lo.2009.54.1.0041.

Mazur, M., Eckley, C. & Mitchell, C. in *European Geosciences Union General Assembly 2013.*

Schuyt, K. & Brander, L. The Economic Values of the World's Wetlands. (WWF-International and Institute for Environmental Studies, Vrije Universiteit, Gland/ Amsterdam, 2004).

Vishnivetskaya, T.A., Mosher, J.J., Palumbo, A.V., Yang, Z.K., Podar, M., Brown, S.D., Brooks, S.C., Gu, B.H., Southworth, G.R., Drake, M.M., Brandt, C.C., and Elias, D.A. (2011). Mercury and Other Heavy Metals Influence Bacterial Community Structure in Contaminated Tennessee Streams. *Applied and Environmental Microbiology* 77**,** 302-311. doi: 10.1128/Aem.01715-10.
